# Investigation of the association between serum uric acid levels and HEART risk score in patients with acute coronary syndrome

**DOI:** 10.14814/phy2.15513

**Published:** 2022-11-16

**Authors:** Ramin Khameneh Bagheri, Mona Najaf Najafi, Mostafa Ahmadi, Mohsen Saberi, Mina Maleki, Vafa Baradaran Rahimi

**Affiliations:** ^1^ Department of Cardiovascular Diseases, Faculty of Medicine Mashhad University of Medical Sciences Mashhad Iran; ^2^ Clinical Research Center, Imam Reza Hospital, Faculty of Medicine Mashhad University of Medical Sciences Mashhad Iran

**Keywords:** acute coronary syndrome, HEART score, uric acid

## Abstract

The association between uric acid (UA) and cardio‐metabolic conditions has been recognized for a long time. However, recently, a body of evidence has highlighted the independent role of UA in a series of conditions, including renal and cardiovascular diseases. In this light, data regarding the prognostic role of UA in acute coronary syndrome (ACS) is scarce. A total number of 100 patients, 59 males and 41 females, diagnosed with ACS were recruited in this study. At the time of admission to the hospital, the serum level of UA was measured. In addition, the HEART score was calculated based on each patients' profile. Participants were on average 61.37 ± 12.08 years old. The most prevalent risk factors were hypertension (48%), a history of coronary artery disease (40%), and diabetes mellitus (33%). The average serum level of UA was 5.81 ± 1.81 mg/dl, and the calculated HEART score had a median of six (minimum of two and maximum of ten). A positive yet statistically insignificant correlation was found between the measured UA level and the calculated HEART score (*R* = 0.375, *p* = 0.090). However, further studies with larger sample size are required to assess the direct association of UA level with major adverse cardiac events in patients with cardiovascular disease.

## Graphical abstract

Our results suggest a modest positive yet statistically insignificant correlation between the serum level of UA and HEART score. However, further studies are needed to determine the exact correlation between UA and HEART score.

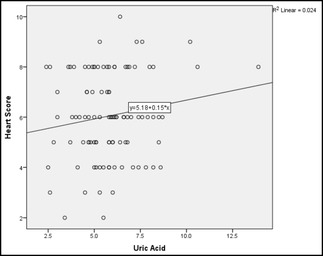

## 1 INTRODUCTION

Worldwide, cardiovascular diseases (CVD), including coronary artery disease (CAD), are the leading cause of death (World Health Organization, 2016). Enhancing the knowledge about predisposing factors of CVD can result in developing prognostic tools and providing early, cost‐effective interventions (Dastani et al., 2022; Gholoobi et al., 2021).

Recently, the role of uric acid (UA) and hyperuricemia as independent risk factors in CAD has regained attention (Ndrepepa, 2018). The xanthine oxidase activity produces UA as the final product of purine catabolism (Richette et al., 2017). While a clear link between the serum level of UA and a series of conditions, including hypertension, chronic kidney injury, diabetes mellitus, obesity, dyslipidemia, and CAD, has been established (Kuwabara et al., 2017; Kuwabara et al., 2018; Li et al., 2017), the exact mechanism has been a subject of debate (El Din et al., 2017; Landolfo & Borghi, 2019).

On the one hand, UA may adversely impact the cardiovascular system through injuring kidneys leading to a pro‐oxidative state and inflammation that induces arteriosclerosis and atherosclerosis (Sánchez‐Lozada et al., 2008). On the other hand, excessive intracellular levels of UA may induce endothelial damage and microvascular dysfunction by reducing nitric oxide levels (Askari et al., 2021; Yu et al., 2010). Despite these possible interpretations, information regarding the association of UA with major adverse cardiovascular events (MACE) is limited. The existing data had resulted from long‐term and mid‐term MACE investigations in patients presenting with myocardial infarction (MI) or acute coronary syndrome (ACS) (Kaya et al., 2012; Lazzeri et al., 2010; Magnoni et al., 2017; Timóteo et al., 2013; Tscharre et al., 2018). As far as we know, no study has yet addressed the association of UA with short‐term MACE in patients with ACS.

In this regard, a widely used prognostic tool to assess the risk of short‐term MACE in patients presenting with non‐specific chest pain is the HEART score (Six et al., 2008). Due to the high sensitivity and specificity of the HEART score for predicting MACE (Van Den Berg & Body, 2018), we used it as an indirect measure. Therefore, we aimed to investigate the association between HEART score and UA serum level in patients diagnosed with ACS.

## 2 METHODS

### 2.1 Ethical statement

In order to conduct this study, approval from the Ethics Committee of Mashhad University of Medical Sciences was obtained (approval code: IR.MUMS.MEDICAL.REC.1398.703). Furthermore, all participants signed an informed written consent form prior to recruitment.

### 2.2 Calculation of sample size

The power (β) was set as 90%, and the significance level (α) was adjusted to 5%. According to a prior study (Erdogan et al., 2005) and the formula for correlation between two parameters (UA and carotid intima‐media thickness, as a predictor for development and/or progression of atherosclerosis), the estimated sample size was 84.

$$N={\left[\left(Z_{1-\frac{\alpha }{2}}+Z_{1-\beta }\right)/C\right]}^{2}+3$$

$$C=0.5^{*}\ln\left[\left(1+r\right)/\left(1-r\right)\right]$$

where $Z_{1-\frac{\alpha }{2}}$ = 1.96, $Z_{1-\beta }$ = 1.28, *r* = 0.346, *C* = 0.36, and *N* = 84. In this regard, 100 patients were included in the current clinical study due to a possible dropout rate of about 20%.

### 2.3 Study design and population

The present cross‐sectional study was conducted on all ACS patients referred to the Ghaem hospital affiliated with Mashhad University of Medical Sciences, Mashhad, Iran, from March to September 2018. ACS patients who fulfilled the American college of cardiology guidelines for both sexes were included in the study. The exclusion criteria were patients who did not complete their treatment course (self‐discharged or transferred to another hospital) or lack of consent.

### 2.4 Data acquisition

The HEART score, widely used as a prognostic marker to assess outcomes in ACS, was calculated for each patient (Table 1). The HEART score is composed of five predictors: History, Electrocardiogram, Age, Risk factors, and Troponin level. Each of these predictors was given points between zero an two based on the extent of the abnormality. History was classified based on narrative records written in patients' charts at admission. In the absence of specific chest pain characteristics, history was classified as “slightly suspicious” and scored zero. If both specific and non‐specific elements were present, it was considered “moderately suspicious” and scored one, and if it contained only specific features, it was labeled as “highly suspicious” and scored two points. ECG findings, interpreted by a cardiologist, were labeled as follows: normal (zero points), with non‐specific repolarization (one point) or significant elevation of ST‐segment (two points). With regard to age, patients younger than 45 scored zero, those between 45 and 65 scored one, and older patients scored two.

**TABLE 1:** The HEART score

Component	Grade	Score
History	Highly suspicious	2
	Moderately suspicious	1
	Slightly or non‐suspicious: absence of specific characteristics of chest pain	0
Electrocardiogram	Significant ST depression	2
	Non‐specific repolarisation disturbance	1
	Normal ECG	0
Age	≥65 years	2
	45–65 years	1
	≤45 years	0
Risk factors	≥3 risk factors^a^ or history of atherosclerotic disease	2
	1 or 2 risk factors	1
	No risk factors known	0
Troponin	≥3× normal limit	2
	1–2× normal limit	1
	≤ normal limit	0

^a^ Risk factors for atherosclerotic disease: Hypercholesterolemia, Cigarette smoking, Hypertension, Positive family history of CAD, Diabetes Mellitus, Obesity (BMI > 30).

In order to count the risk factors, a checklist including the following items was completed for each patient: history of diagnosed hypertension, diabetes mellitus, hypercholesterolemia, coronary artery disease, family history of coronary artery disease (parent or sibling with CAD before age 65), and smoking (current or cessation less than 1 month). Body mass index (BMI) was calculated using recorded height and weight; BMI > 30 was accounted as a risk factor. For patients with no risk factors, no points were given. Those with one or two risk factors were given one point, and those with more than two risk factors were assigned two points.

Finally, the troponin I level was assessed: zero points were given if the Troponin level was within the normal range (<0.04 ng/ml), one point was given if it was one to three times of normal limit, and two points were assigned if it was more than three times of normal limit. The calculated Heart score was classified into three intervals: interval one with scores between zero and three, interval two with scores between four and six, and interval three with scores between seven and ten.

In addition, a blood serum sample was obtained from patients at the time of admission in order to measure the level of UA (mg/dl). The UA level was used as an independent factor in the analyses.

### 2.5 Statistics

Data were analyzed using the SPSS version. 23.0 statistical software (SPSS Inc., Chicago, Illinois). Data distribution was assessed using the One‐sample Kolmogorov–Smirnov test. Descriptive data were reported as mean ± standard deviation (SD) and median (interquartile range) for normally and non‐normally distributed data, respectively. As appropriate, the comparison between quantitative variables was performed using parametric one‐way analysis of variances (ANOVA) for normally distributed data or the Kruskal–Wallis test for the non‐normally distributed variables. In addition, for comparing the qualitative variables, the Chi‐squared test was used. Spearman's correlation coefficient was used to assess the association between the HEART score and UA level. Statistical significance was defined as *p* < 0.05 for all the statistical tests.

## 3 RESULTS

Overall, 100 patients, 59 males and 41 females, were recruited in the study from 20th March to 20th September 2018. The mean age of patients was 61.37 ± 12.08 years old, and the calculated BMI was 24.31 ± 2.54 kg/m^2^. Our results revealed that 48 (48.0%) patients had hypertension, 33 (33.0%) patients had Diabetes Mellitus, 29 (29.0%) had hypercholesterolemia, and 40 (40.0%) patients had a history of CAD. Moreover, 23 (23.0%) patients were smokers, and 17 (17.0%) had a positive family history of CAD. The HEART score was with a median of six, a minimum of two, and a maximum of 10, and the average serum level of UA was 5.81 ± 1.81 mg/dl among the enrolled patients.

**FIGURE 1:**
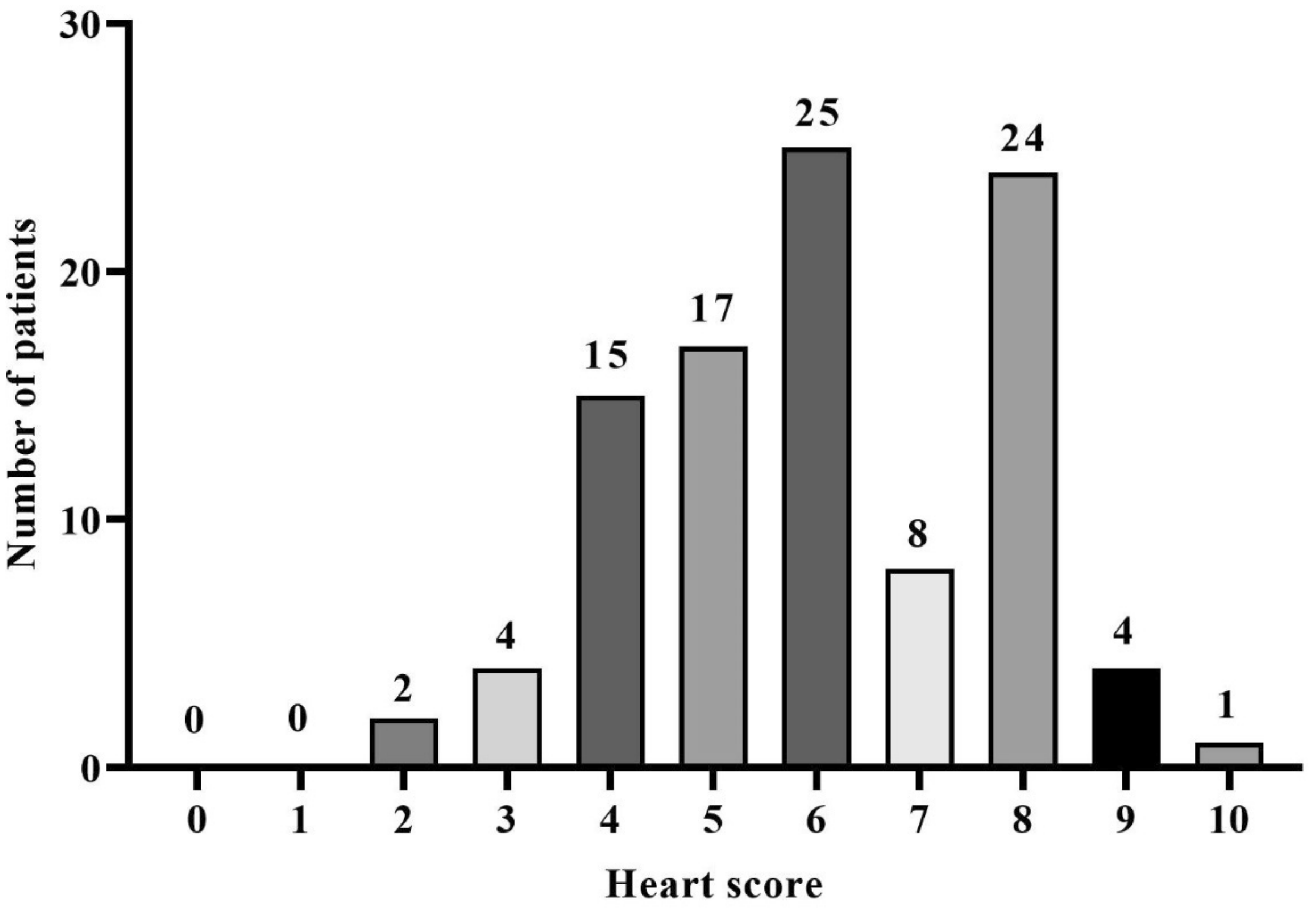
The HEART score frequency between the enrolled patients.

The HEART score frequency between the enrolled patients is shown in Figure 1. For further analysis, patients were classified into three groups based on their HEART score: 6 (6.0%) patients were in group 1 with scores of 0–3, 57 (57.0%) in group 2 with scores of 4–6 and 37 (37.0%) in group 3 with scores of 7–10 (Table 2). Detailed descriptive data according to the HEART score is illustrated in Table 2. Our results showed that patients with the higher HEART score had higher age (*p* = 0.002), higher prevalence of hypertension (*p* = 0.047), highly suspicious for ACS (*p* = 0.008), significant elevation of ST‐segment (*p* < 0.001), and higher troponin levels (*p* < 0.001, Table 2). In addition, no significant differences were observed in the serum UA level between the three HEART score groups (*p* = 0.150, Table 2).

**TABLE 2:** Comparison of study variables between three classes of the HEART score

Variables	HEART score			Total (*N* = 100)	*p*‐value
	0–3 (*N* = 6)	4–6 (*N* = 57)	7–10 (*N* = 37)		
Male (*n*, %)	2 (33.3%)	32 (56.1%)	25 (67.5%)	59 (59.0%)	0.299^a^
Female (*n*, %)	4 (66.6%)	25 (43.9%)	12 (32.5%)	41 (41.0%)	
Age (years, Mean ± SD)	55.67 ± 5.16	58.42 ± 11.71	66.84 ± 11.59	61.37 ± 12.08	0.002^b^
Diabetes Mellitus (*n*, %)	1 (16.6%)	21 (36.8%)	11 (29.7%)	33 (33.0%)	0.526^a^
Hypertension (*n*, %)	1 (16.6%)	24 (42.1%)	33 (89.2%)	58 (58.0%)	0.047^a^
Hypercholesterolemia (*n*, %)	3 (50.0%)	16 (28.0%)	10 (27.0%)	29 (29.0%)	0.502^a^
CAD (*n*, %)	0 (0.0%)	24 (42.1%)	16 (43.2%)	40 (40.0%)	0.118^a^
Family history of CAD (*n*, %)	0 (0.0%)	13 (22.8%)	4 (10.8%)	17 (17.0%)	0.166^a^
Smoking (*n*, %)	0 (0.0%)	15 (26.3%)	8 (21.6%)	23 (23.0%)	0.335^a^
BMI (kg/m^2^, Mean ± SD)	24.02 ± 2.0	24.18 ± 2.26	24.54 ± 3.02	24.31 ± 2.54	0.768^b^
History of chest pain					
Slightly suspicious (0 points, *n*, %)	1 (16.6%)	6 (10.5%)	2 (5.4%)	9 (9.0%)	0.008^a^
Moderately suspicious (1 point, *n*, %)	4 (66.8%)	22 (38.6%)	5 (13.5%)	31 (31.0%)	
Highly suspicious (2 points, *n*, %)	1 (16.6%)	29 (50.9%)	30 (81.1%)	60 (60.0%)	
ECG interpretation					
Normal (*n*, %)	6 (100.0%)	27 (47.4%)	1 (2.7%)	34 (34.0%)	<0.001^a^
Non‐specific repolarization (*n*, %)	0 (0.0%)	10 (17.5%)	6 (16.2%)	16 (16.0%)	
Significant Elevation of ST segment (*n*, %)	0 (0.0%)	20 (35.1%)	30 (81.1%)	50 (50.0%)	
Lab data					
Troponin (ng/ml) Median (interquartile range)	0.01 (0.00–0.32)	0.01 (0.00–0.50)	0.91 (0.00–0.5)	0.31 (0.00–0.5)	<0.001^b^
UA (mg/dl) Median (Interquartile range)	4.9 (2.6–6.0)	5.9 (2.5–8.7)	5.7 (2.4–13.9)	5.81 ± 1.81	0.150^b^

Abbreviations: BMI, Body mass index; CAD, Coronary artery disease.

^a^ Chi‐square test was used.

^b^ One‐way ANOVA or Kruskal–Wallis was performed.

**FIGURE 2:**
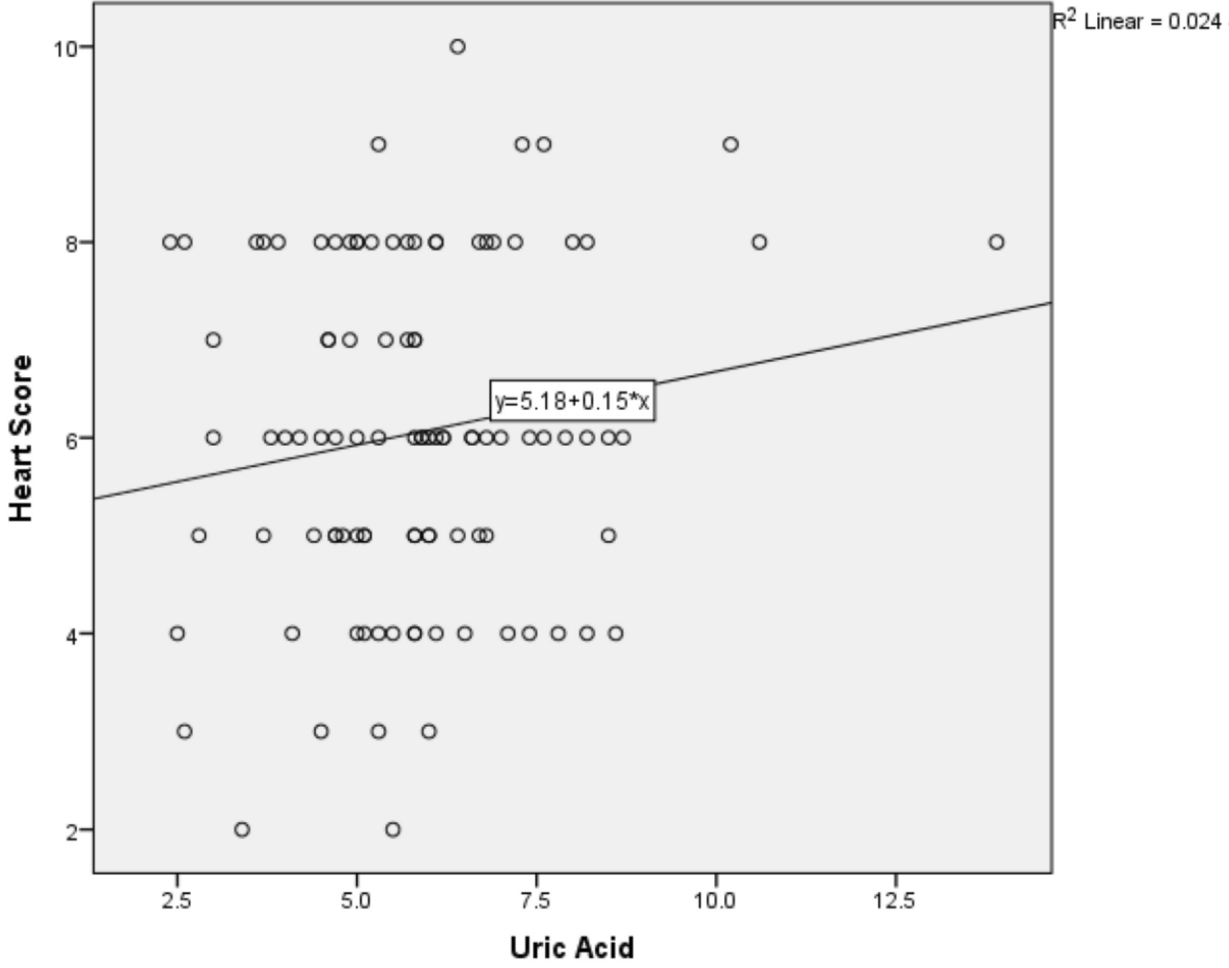
The correlation analysis between the UA level and HEART score (correlation coefficient = 0.375, *p* = 0.090).

The average serum level of UA was 5.81 ± 1.81 mg/dl among the enrolled patients. In order to assess the association between UA level and HEART score, a Spearman correlation analysis was performed, which resulted in a positive but insignificant correlation coefficient of 0.375 (*p* = 0.090). Linear regression analysis also yielded a similar positive insignificant association (Figure 2).

## 4 DISCUSSION

Predicting the risk of short‐term adverse outcomes in the setting of acute coronary syndrome is crucial for accurate and efficient decision‐making. Therefore, it is necessary to recognize the risk factors that influence the course of the disease. In order to shed light on the role of UA as a risk factor in acute coronary syndrome, we investigated the relationship between UA and HEART score in patients diagnosed with ACS. To the best of our knowledge, this is the first study evaluating this question.

We found a modest positive yet statistically insignificant correlation (0.375, *p* = 0.090) between the serum level of UA and HEART score. Considering other analyzed variables, we only detected a significant positive correlation between age and hypertension as a risk factor and the HEART score.

Inconsistent with our findings, UA has been associated with multiple interrelated conditions, including hypertension, glucose intolerance, overweight and dyslipidemia (metabolic syndrome), renal diseases, and cardiovascular diseases (El Din et al., 2017; Li et al., 2017). In this regard, there has been uncertainty about the causality of the observed association; whether UA is merely a biological marker or has an independent prognostic value is important, mainly because its serum level can easily be reduced using proper available treatments. Recent findings have provided a large body of evidence supporting the independent causal role of UA (El Din et al., 2017). Kuwabara et al. conducted a five‐year cohort study in Japan and reported that asymptomatic hyperuricemia in the absence of comorbidities (healthy adults) was associated with a higher risk for developing hypertension, dyslipidemia, chronic kidney disease, and overweight/obesity (Kuwabara et al., 2017). It has also been shown that treatment with allopurinol decreases insulin resistance (Takir et al., 2015), body weight, and blood pressure (Madero et al., 2015; Soletsky & Feig, 2012).

Few yet consistent studies assessed the relationship between UA and MACE outcomes concerning CVD. Two studies reported that a higher level of UA was associated with in‐hospital mortality and adverse outcomes in patients with MI undergoing percutaneous coronary intervention (PCI) (Kaya et al., 2012; Lazzeri et al., 2010). Tscharre and colleagues asked the same question in a more diverse group of patients diagnosed with ACS undergoing PCI. They found that hyperuricemia was linked to a 1.6‐fold increase in long‐term MACE (Tscharre et al., 2018). In contrast to mentioned studies that measured long‐term outcomes, Magnoni et al. (2017), Pagidipati et al. (2017), and Timóteo et al. (2013) evaluated mid‐term (1 year) MACE outcomes in patients diagnosed with ACS and found the same results, higher UA level was associated with a higher incidence of in‐hospital 1‐year mortality.

These findings are further supported by evidence indicative of the role of UA in endothelial damage through inducing oxidative stress (Sánchez‐Lozada et al., 2012; Yu et al., 2010), vascular inflammation caused by overexpression of chemokines (Kanellis et al., 2003; Spiga et al., 2017), and reduction in nitric oxide availability (Gersch et al., 2008). In addition, there are reports of the association between UA and coronary plaques (Saito et al., 2015), carotid plaques (Li et al., 2015), the extent of coronary atherosclerosis, reduced coronary flow reserve, and impaired coronary microvascular function (Akpek et al., 2011; Erdogan et al., 2006; Kaya et al., 2010). Overall, it seems reasonably justified to see and use UA to anticipate the mid‐ to long‐term MACE outcomes in patients with CAD. However, it is still unclear whether it is of value in detecting and prognosis of short‐term outcomes in acute settings of ACS.

Meanwhile, the HEART score is a new but repeatedly validated prognostic tool to estimate the risk of short‐term MACE outcomes (6 weeks) in patients with non‐specific chest pain or non‐ST‐elevation presentation of ACS (Six et al., 2008). It comprises of five classes of items; risk factors and age are relatively static components, whereas history, ECG pattern, and troponin I level are dynamic. It has been demonstrated that a low HEART score (0–3) is associated with a relatively low risk of adverse events (1.6%) in the next 6 weeks (Van Den Berg & Body, 2018), while moderate (Kuwabara et al., 2017; Richette et al., 2017; Ndrepepa, 2018) and high score (El Din et al., 2017; Kuwabara et al., 2018; Landolfo & Borghi, 2019; Li et al., 2017) comprise a higher risk of 11.6% and 65.2%, respectively (Backus et al., 2010). Consequently, our observation of the comparative change in the distribution of UA, rise in median, and expansion of interquartile range along the HEART score bands suggests a possible prognostic role for UA in acute cardiovascular events.

Our study was designed as a pilot study to postulate the role of UA in acute cardiovascular events and assess its relation with the HEART score. The main limitation of this study was its indirect approach toward short‐term MACE outcomes. In order to accurately evaluate the prognostic role of UA, the endpoints (i.e., MACE outcomes) should be clearly defined and recorded. Then it will be possible to compare the predictive value of the HEART score and UA and ponder whether the addition of the serum level of UA to the HEART score system would increase the precision of anticipating short‐term MACE outcomes or not. Additionally, we evaluated 100 patients with ACS, and it is necessary to perform further studies with a larger sample size. Furthermore, the role of treatment with allopurinol to reduce UA and consequently prevent undesired cardiovascular events is another interesting subject to investigate.

## 5 CONCLUSION

In acute coronary settings, making accurate and cost‐effective decisions is critical. Thus, the most important factors should be incorporated to assess the situation better and predict the outcomes. While the HEART score is already in use for this purpose, the existing literature also suggests a promising role for UA. In fact, our results suggest a modest positive yet statistically insignificant correlation between the serum level of UA and the HEART score. However, further studies with a larger sample size are needed to determine the exact correlation between UA and HEART score.

## AUTHORS' CONTRIBUTIONS

Ramin Khameneh Bagheri: Conceptualisation, Methodology, Funding Acquisition, Investigation; Mona Najaf Najafi: Formal Analysis, Data Curation; Mostafa Ahmadi: Conceptualisation, Methodology, Investigation; Mohsen Saberi: Investigation, Data Curation; Mina Maleki: Investigation, Data Curation, Writing—Original Draft, Vafa Baradaran Rahimi: Formal Analysis, Writing—Original Draft, Writing—review & editing.

## CONFLICT OF INTEREST

The authors declare no conflict of interest.

## Data Availability

The datasets used for the current study are available from the corresponding author on reasonable request.

## References

[phy215513-bib-0001] Akpek, M. , Kaya, M. G. , Uyarel, H. , Yarlioglues, M. , Kalay, N. , Gunebakmaz, O. , Dogdu, O. , Ardic, I. , Elcik, D. , Sahin, O. , Oguzhan, A. , Ergin, A. , & Gibson, C. M. (2011). The association of serum uric acid levels on coronary flow in patients with STEMI undergoing primary PCI. Atherosclerosis, 219(1), 334–341. 21831375 10.1016/j.atherosclerosis.2011.07.021

[phy215513-bib-0002] Askari, V. R. , Rahimi, V. B. , Zargarani, R. , Ghodsi, R. , Boskabady, M. , & Boskabady, M. H. (2021). Anti‐oxidant and anti‐inflammatory effects of auraptene on phytohemagglutinin (PHA)‐induced inflammation in human lymphocytes. Pharmacological Reports, 73(1), 154–162. 32166733 10.1007/s43440-020-00083-5

[phy215513-bib-0003] Backus, B. E. , Six, A. J. , Kelder, J. C. , Mast, T. P. , van den Akker, F. , Mast, E. G. , Monnink, S. H. J. , van Tooren, R. M. , & Doevendans, P. A. F. M. (2010). Chest pain in the emergency room: A multicenter validation of the HEART score. Critical Pathways in Cardiology, 9(3), 164–169. 20802272 10.1097/HPC.0b013e3181ec36d8

[phy215513-bib-0004] Dastani, M. , Rahimi, H. R. , Askari, V. R. , Jaafari, M. R. , Jarahi, L. , Yadollahi, A. , & Rahimi, V. B. (2022). Three months of combination therapy with nano‐curcumin reduces the inflammation and lipoprotein (a) in type 2 diabetic patients with mild to moderate coronary artery disease: Evidence of a randomized, double‐blinded, placebo‐controlled clinical trial. BioFactors. Epub ahead of print. 10.1002/biof.1874 35674733

[phy215513-bib-0005] El Din, U. A. S. , Salem, M. M. , & Abdulazim, D. O. (2017). Uric acid in the pathogenesis of metabolic, renal, and cardiovascular diseases: A review. Journal of Advanced Research, 8(5), 537–548. 28748119 10.1016/j.jare.2016.11.004 PMC5512153

[phy215513-bib-0006] Erdogan, D. , Gullu, H. , Caliskan, M. , Yildirim, E. , Bilgi, M. , Ulus, T. , Sezgin, N. , & Muderrisoglu, H. (2005). Relationship of serum uric acid to measures of endothelial function and atherosclerosis in healthy adults. International Journal of Clinical Practice, 59(11), 1276–1282. 16236080 10.1111/j.1742-1241.2005.00621.x

[phy215513-bib-0007] Erdogan, D. , Gullu, H. , Caliskan, M. , Yildirim, I. , Ulus, T. , Bilgi, M. , Yilmaz, S. , & Muderrisoglu, H. (2006). Coronary flow reserve and coronary microvascular functions are strongly related to serum uric acid concentrations in healthy adults. Coronary Artery Disease, 17(1), 7–14. 16374135 10.1097/00019501-200602000-00002

[phy215513-bib-0008] Gersch, C. , Palii, S. P. , Kim, K. M. , Angerhofer, A. , Johnson, R. J. , & Henderson, G. N. (2008). Inactivation of nitric oxide by uric acid. Nucleosides, Nucleotides and Nucleic Acids, 27(8), 967–978. 10.1080/15257770802257952 PMC2701227 18696365

[phy215513-bib-0009] Gholoobi, A. , Askari, V. R. , Naghedinia, H. , Ahmadi, M. , Vakili, V. , & Baradaran Rahimi, V. (2021). Colchicine effectively attenuates inflammatory biomarker high‐sensitivity C‐reactive protein (hs‐CRP) in patients with non‐ST‐segment elevation myocardial infarction: A randomised, double‐blind, placebo‐controlled clinical trial. Inflammopharmacology, 29(5), 1379–1387. 34420187 10.1007/s10787-021-00865-0

[phy215513-bib-0010] Kanellis, J. , Watanabe, S. , Li, J. H. , Kang, D. H. , Li, P. , Nakagawa, T. , Wamsley, A. , Sheikh‐Hamad, D. , Lan, H. Y. , Feng, L. , & Johnson, R. J. (2003). Uric acid stimulates monocyte chemoattractant protein‐1 production in vascular smooth muscle cells via mitogen‐activated protein kinase and cyclooxygenase‐2. Hypertension, 41(6), 1287–1293. 12743010 10.1161/01.HYP.0000072820.07472.3B

[phy215513-bib-0011] Kaya, E. B. , Yorgun, H. , Canpolat, U. , Hazırolan, T. , Sunman, H. , Ülgen, A. , Ates, A. H. , Aytemir, K. , Tokgözoğlu, L. , Kabakcı, G. , Akata, D. , & Oto, A. (2010). Serum uric acid levels predict the severity and morphology of coronary atherosclerosis detected by multidetector computed tomography. Atherosclerosis, 213(1), 178–183. 20863499 10.1016/j.atherosclerosis.2010.08.077

[phy215513-bib-0012] Kaya, M. G. , Uyarel, H. , Akpek, M. , Kalay, N. , Ergelen, M. , Ayhan, E. , Isik, T. , Cicek, G. , Elcik, D. , Sahin, Ö. , Cosgun, S. M. , Oguzhan, A. , Eren, M. , & Gibson, C. M. (2012). Prognostic value of uric acid in patients with ST‐elevated myocardial infarction undergoing primary coronary intervention. The American Journal of Cardiology, 109(4), 486–491. 22100027 10.1016/j.amjcard.2011.09.042

[phy215513-bib-0013] Kuwabara, M. , Borghi, C. , Cicero, A. F. G. , Hisatome, I. , Niwa, K. , Ohno, M. , Johnson, R. J. , & Lanaspa, M. A. (2018). Elevated serum uric acid increases risks for developing high LDL cholesterol and hypertriglyceridemia: A five‐year cohort study in Japan. International Journal of Cardiology, 261, 183–188. 29551256 10.1016/j.ijcard.2018.03.045

[phy215513-bib-0014] Kuwabara, M. , Niwa, K. , Hisatome, I. , Nakagawa, T. , Roncal‐Jimenez, C. A. , Andres‐Hernando, A. , Bjornstad, P. , Jensen, T. , Sato, Y. , Milagres, T. , Garcia, G. , Ohno, M. , Lanaspa, M. A. , & Johnson, R. J. (2017). Asymptomatic hyperuricemia without comorbidities predicts cardiometabolic diseases: Five‐year Japanese cohort study. Hypertension, 69(6), 1036–1044. 28396536 10.1161/HYPERTENSIONAHA.116.08998 PMC5426964

[phy215513-bib-0015] Landolfo, M. , & Borghi, C. (2019). Hyperuricaemia and vascular risk: The debate continues. Current Opinion in Cardiology, 34(4), 399–405. 30925517 10.1097/HCO.0000000000000626

[phy215513-bib-0016] Lazzeri, C. , Valente, S. , Chiostri, M. , Sori, A. , Bernardo, P. , & Gensini, G. F. (2010). Uric acid in the acute phase of ST elevation myocardial infarction submitted to primary PCI: Its prognostic role and relation with inflammatory markers: A single center experience. International Journal of Cardiology, 138(2), 206–209. 18684529 10.1016/j.ijcard.2008.06.024

[phy215513-bib-0017] Li, Q. , Zhou, Y. , Dong, K. , Wang, A. , Yang, X. , Zhang, C. , Zhu, Y. , Wu, S. , & Zhao, X. (2015). The association between serum uric acid levels and the prevalence of vulnerable atherosclerotic carotid plaque: A cross‐sectional study. Scientific Reports, 5(1), 1–6. 10.1038/srep10003 PMC4426733 25961501

[phy215513-bib-0018] Li, X. , Meng, X. , Timofeeva, M. , Tzoulaki, I. , Tsilidis, K. K. , Ioannidis, J. P. , Campbell, H. , & Theodoratou, E. (2017). Serum uric acid levels and multiple health outcomes: Umbrella review of evidence from observational studies, randomised controlled trials, and mendelian randomisation studies. BMJ, 357, j2376. 28592419 10.1136/bmj.j2376 PMC5461476

[phy215513-bib-0019] Madero, M. , Rodríguez Castellanos, F. E. , Jalal, D. , Villalobos‐Martín, M. , Salazar, J. , Vazquez‐Rangel, A. , Johnson, R. J. , & Sanchez‐Lozada, L. G. (2015). A pilot study on the impact of a low fructose diet and allopurinol on clinic blood pressure among overweight and prehypertensive subjects: A randomized placebo controlled trial. Journal of the American Society of Hypertension, 9(11), 837–844. 26329473 10.1016/j.jash.2015.07.008

[phy215513-bib-0020] Magnoni, M. , Berteotti, M. , Ceriotti, F. , Mallia, V. , Vergani, V. , Peretto, G. , Angeloni, G. , Cristell, N. , Maseri, A. , & Cianflone, D. (2017). Serum uric acid on admission predicts in‐hospital mortality in patients with acute coronary syndrome. International Journal of Cardiology, 240, 25–29. 28476518 10.1016/j.ijcard.2017.04.027

[phy215513-bib-0021] Ndrepepa, G. (2018). Uric acid and cardiovascular disease. Clinica Chimica Acta, 484, 150–163. 10.1016/j.cca.2018.05.046 29803897

[phy215513-bib-0022] Pagidipati, N. J. , Hess, C. N. , Clare, R. M. , Akerblom, A. , Tricoci, P. , Wojdyla, D. , Keenan, R. T. , James, S. , Held, C. , Mahaffey, K. W. , Klein, A. B. , Wallentin, L. , & Roe, M. T. (2017). An examination of the relationship between serum uric acid level, a clinical history of gout, and cardiovascular outcomes among patients with acute coronary syndrome. American Heart Journal, 187, 53–61. 28454808 10.1016/j.ahj.2017.02.023 PMC9806969

[phy215513-bib-0023] Richette, P. , Doherty, M. , Pascual, E. , Barskova, V. , Becc, F. , Castañeda‐Sanabria, J. , Coyfish, M. , Guillo, S. , Jansen, T. L. , Janssens, H. , Lioté, F. , Mallen, C. , Nuki, G. , Perez‐Ruiz, F. , Pimentao, J. , Punzi, L. , Pywell, T. , So, A. , Tausche, A. K. , … Bardin, T. (2017). 2016 updated EULAR evidence‐based recommendations for the management of gout. Annals of the Rheumatic Diseases, 76, 29–42. 27457514 10.1136/annrheumdis-2016-209707

[phy215513-bib-0024] Saito, Y. , Nakayama, T. , Sugimoto, K. , Fujimoto, Y. , & Kobayashi, Y. (2015). Relation of lipid content of coronary plaque to level of serum uric acid. The American Journal of Cardiology, 116(9), 1346–1350. 26381534 10.1016/j.amjcard.2015.07.059

[phy215513-bib-0025] Sánchez‐Lozada, L. G. , Lanaspa, M. A. , Cristóbal‐García, M. , García‐Arroyo, F. , Soto, V. , Cruz‐Robles, D. , Nakagawa, T. , Yu, M. A. , Kang, D. H. , & Johnson, R. J. (2012). Uric acid‐induced endothelial dysfunction is associated with mitochondrial alterations and decreased intracellular ATP concentrations. Nephron Experimental Nephrology, 121(3–4), e71–e78. 23235493 10.1159/000345509 PMC3656428

[phy215513-bib-0026] Sánchez‐Lozada, L. G. , Soto, V. , Tapia, E. , Avila‐Casado, C. , Sautin, Y. Y. , Nakagawa, T. , Franco, M. , Rodríguez‐Iturbe, B. , & Johnson, R. J. (2008). Role of oxidative stress in the renal abnormalities induced by experimental hyperuricemia. American Journal of Physiology ‐ Renal Physiology, 295(4), F1134–F1141. 18701632 10.1152/ajprenal.00104.2008 PMC2576157

[phy215513-bib-0027] Six, A. , Backus, B. , & Kelder, J. (2008). Chest pain in the emergency room: Value of the HEART score. Netherlands Heart Journal, 16(6), 191–196. 18665203 10.1007/BF03086144 PMC2442661

[phy215513-bib-0028] Soletsky, B. , & Feig, D. I. (2012). Uric acid reduction rectifies prehypertension in obese adolescents. Hypertension, 60(5), 1148–1156. 23006736 10.1161/HYPERTENSIONAHA.112.196980

[phy215513-bib-0029] Spiga, R. , Marini, M. A. , Mancuso, E. , di Fatta, C. , Fuoco, A. , Perticone, F. , Andreozzi, F. , Mannino, G. C. , & Sesti, G. (2017). Uric acid is associated with inflammatory biomarkers and induces inflammation via activating the NF‐κB signaling pathway in HepG2 cells. Arteriosclerosis, Thrombosis, and Vascular Biology, 37(6), 1241–1249. 28408375 10.1161/ATVBAHA.117.309128

[phy215513-bib-0030] Takir, M. , Kostek, O. , Ozkok, A. , Elcioglu, O. C. , Bakan, A. , Erek, A. , Mutlu, H. H. , Telci, O. , Semerci, A. , Odabas, A. R. , Afsar, B. , Smits, G. , ALanaspa, M. , Sharma, S. , Johnson, R. J. , & Kanbay, M. (2015). Lowering uric acid with allopurinol improves insulin resistance and systemic inflammation in asymptomatic hyperuricemia. Journal of Investigative Medicine, 63(8), 924–929. 26571421 10.1097/JIM.0000000000000242

[phy215513-bib-0031] Timóteo, A. T. , Lousinha, A. , Labandeiro, J. , Miranda, F. , Papoila, A. L. , Oliveira, J. A. , Ferreira, M. L. , & Ferreira, R. C. (2013). Serum uric acid: A forgotten prognostic marker in acute coronary syndromes? European Heart Journal Acute Cardiovascular Care, 2(1), 44–52. 24062933 10.1177/2048872612474921 PMC3760573

[phy215513-bib-0032] Tscharre, M. , Herman, R. , Rohla, M. , Hauser, C. , Farhan, S. , Freynhofer, M. K. , Huber, K. , & Weiss, T. W. (2018). Uric acid is associated with long‐term adverse cardiovascular outcomes in patients with acute coronary syndrome undergoing percutaneous coronary intervention. Atherosclerosis, 270, 173–179. 29432935 10.1016/j.atherosclerosis.2018.02.003

[phy215513-bib-0033] Van Den Berg, P. , & Body, R. (2018). The HEART score for early rule out of acute coronary syndromes in the emergency department: A systematic review and meta‐analysis. European Heart Journal Acute Cardiovascular Care, 7(2), 111–119. 28534694 10.1177/2048872617710788

[phy215513-bib-0034] World Health Organization . (2016). World health statistics 2016: Monitoring health for the SDGs sustainable development goals. World Health Organization.

[phy215513-bib-0035] Yu, M.‐A. , Sánchez‐Lozada, L. G. , Johnson, R. J. , & Kang, D. H. (2010). Oxidative stress with an activation of the renin–angiotensin system in human vascular endothelial cells as a novel mechanism of uric acid‐induced endothelial dysfunction. Journal of Hypertension, 28(6), 1234–1242. 20486275

